# Predictive Value of *KDM5C* Alterations for Immune Checkpoint Inhibitors Treatment Outcomes in Patients With Cancer

**DOI:** 10.3389/fimmu.2021.664847

**Published:** 2021-04-19

**Authors:** Xiao-Juan Chen, Ai-Qun Ren, Liang Zheng, En-Dian Zheng

**Affiliations:** ^1^ Department of Clinical Medicine, Graduate School, Zhejiang Chinese Medical University, Hangzhou, China; ^2^ Department of Gastroenterology, Wenzhou People’s Hospital, Wenzhou Third Clinical Institute Affiliated to Wenzhou Medical University, Wenzhou, China

**Keywords:** immune checkpoint inhibitors, biomarker, *KDM5C*, outcome, prognosis

## Abstract

Lysine (K)-specific demethylase 5C (*KDM5C*) plays a significant role in the tumor cell proliferation, invasion, drug resistance and the regulation of tumor-related gene expression. Here, we aimed to investigate its predictive value in patients with cancers received immune checkpoint inhibitors (ICIs). We explored the predictive value of *KDM5C* alterations and the association between *KDM5C* alteration and immune landscape by using published cohort with clinical outcome and sequenced data from online database. The frequency of *KDM5C* alterations was 2.1% across 48045 tumor samples with different cancers from 185 studies. *KDM5C* alterations were correlated with markedly inferior overall survival (OS, 53 vs. 102 months, *P*<0.0001) than those without. However, in ICI-treated group, patients with *KDM5C* alterations had a substantially prolonged OS than the wild-type group (not reached vs. 18 months, *P*=0.0041). The predictive value of *KDM5C* alterations for ICI treatment outcome was not observed in patients with microsatellite-stable tumors (*P*=0.2875). Intriguingly, patients with non-small-cell lung cancer and *KDM5C* alterations receiving ICI had the better progression-free survival than wild type group (13.2 vs. 3.2 months, *P*=0.0762). Mechanistically, *KDM5C* altered tumors had dramatically higher TMB level and was associated with significantly higher level of CD8+ T cell infiltration and T effector signature. In conclusion, *KDM5C* alterations was correlated with enhanced tumor immunogenicity and inflamed anti-tumor immunity, thus resulting in better treatment outcome in cancer patients receiving ICIs.

## Introduction

Immune checkpoint inhibitors (ICIs) targeting cytotoxic T lymphocyte antigen-4 (CTLA-4), or programmed cell death protein 1 (PD-1) and its ligand (PD-L1) interaction have shifted the treatment paradigms and significantly improve the overall survival (OS) in diverse cancers (1–4). Nevertheless, ICIs could only benefit a minority (~20%) of unselected population (5). Herein, there is an urgent need to develop novel predictive biomarkers for the majority of patients, who could not benefit from ICIs treatment. The mutational landscape of tumor cells is a direct reflection of tumor immunogenicity and could dictate the extent and phenotype of immune infiltrates (6–8). Understanding the relationship between tumor genomic alterations and response to ICIs could lay a foundation for the development of novel predictive biomarkers and therapeutic strategies to improve the clinical benefit (8).

Lysine (K)-specific demethylase 5C (*KDM5C*) is a histone demethylase that specifically removes methyl residues from tri-, di-, and monomethylated lysine 4 on histone H3 lysine 4 (H3K4), thus resulting in suppressing gene transcription by reducing H3K4 trimethylation levels (9–11). Previous studies reported that genetic alterations of *KDM5C* were common in various types of cancers including breast, colon, ovarian, prostate cancer and so on. It plays a significant role in the tumorigenesis, cancer cell proliferation, invasion, drug resistance and the regulation of tumor-related gene expression (12–14). Moreover, a recent elegant study analyzed the multi-omics data of 823 advanced renal cell carcinoma and found that somatic mutations in *KDM5C* correlate with high angiogenesis and AMPK/fatty acid oxidation gene expression, which was enriched in ICIs beneficial group. These findings revealed the contribution of *KDM5C* to antitumor immune response. Therefore, it is valuable to explore the predictive value of *KDM5C* alterations for ICIs treatment outcome in multiple cancers.

Here, we performed this pan-cancer analysis to investigate *KDM5C* alterations frequency and their predictive significance for ICIs treatment outcomes across cancer types. We also evaluated the relationship between *KDM5C* alteration and immune infiltrates and signatures by using online database to unravel the potential mechanism.

## Materials and Methods

### Data Collection and Pan-Cancer Analysis

**Figure 1:**
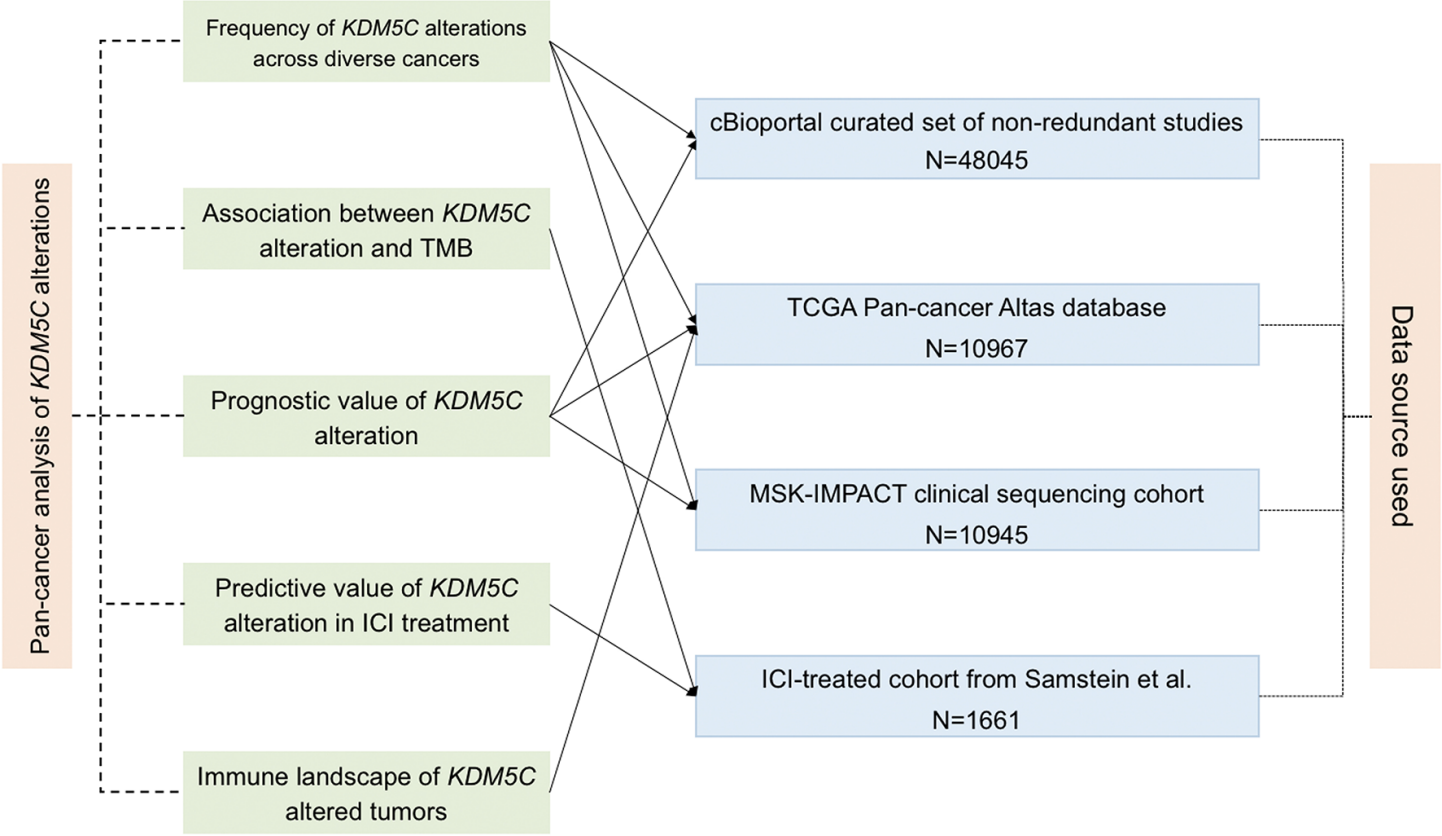
Flowchart of the sequenced data and clinical cohort. The connected solid line between analysis aim (middle left) and data source (middle right) means the used cohort by this analysis.

We downloaded the sequenced data and collected clinical information from several online database as shown in **Figure 1**. For determination of the frequency of *KDM5C* alterations among different types of solid tumors, the genomic alterations and clinical characteristics were identified from the cBioPortal online database (https://www.cbioportal.org) (15, 16). *KDM5C* alterations were recorded as all kinds of nonsynonymous mutations including mutations, missense, frame-shift, splice site, nonstop, nonsense, and translation start site changes. Non-redundant publications were identified. If two or more studies reported the same cohort, only the study with the largest sample size and latest information was included. To avoid the selection bias and limitation of small sample size, we excluded the records of cancer type with patients less than 100. Analysis of TMB normalization, clinical cohort and treatment outcomes were summarized in Supplemental Material.

## Tumor Immunogenicity and Immune Landscape Analysis

To delineate the immune microenvironment features of tumors with *KDM5C* alterations, we calculated and compared immune infiltrates, immune signatures and immune-related gene expression between *KDM5C* altered and wild type group by using RNA-seq data from TCGA. The correlation between *KDM5C* expression and immune checkpoints expression in different cancers was evaluated by using online database, named Tumor Immune Estimation Resource (TIMER). The statistical methods were listed in this website (https://cistrome.shinyapps.io/timer/) and their previous publications (17, 18). The abundance of tumor infiltrating leukocytes, including CD8^+^ T cells, CD4^+^ T cells, regulatory T cells (Tregs), dendritic cells, B cells, macrophage, myeloid-derived suppressor cells (MDSC), NK cells, mast cell, neutrophils, endothelial cells and cancer-associated fibroblasts (CAFs), was estimated by using different bioinformatic algorithm and compared between *KDM5C* altered and wild type group.

### Statistical Analysis

The association between *KDM5C* status and clinical features were evaluated by using fisher’s exact test. χ2 test were performed to test whether the sampling distribution was equal for two groups. The continuous variables were analyzed by ANOVA and Tukey’s multiple comparison tests. The differences of TMB, tumor-infiltrating immune cells, immune signatures, or immune-related gene expressions between *KDM5C* altered and wild type tumors were tested by using Mann-Whitney U test. We conducted two-tailed Mann-Whitney U tests for comparison of the nonparametric data set. Survival outcomes were measured with OS, or progression-free survival (PFS) according to the accessibility for each cohort. Kaplan-Meier curves with two-sided log-rank tests and Cox proportional hazards model with calculated hazard ratios (HRs) and 95% confidence intervals (CIs) were adjusted for available confounding factors to determine the different clinical outcomes between *KDM5C* altered and wild-type groups. Two-sided *P*<0.05 was considered significant. All statistical analyses were performed using the SPSS statistical software, version 20.0 (SPSS Inc., Chicago, IL, USA).

## Results

### Overview of Pan-Cancer Analysis

**Figure 2:**
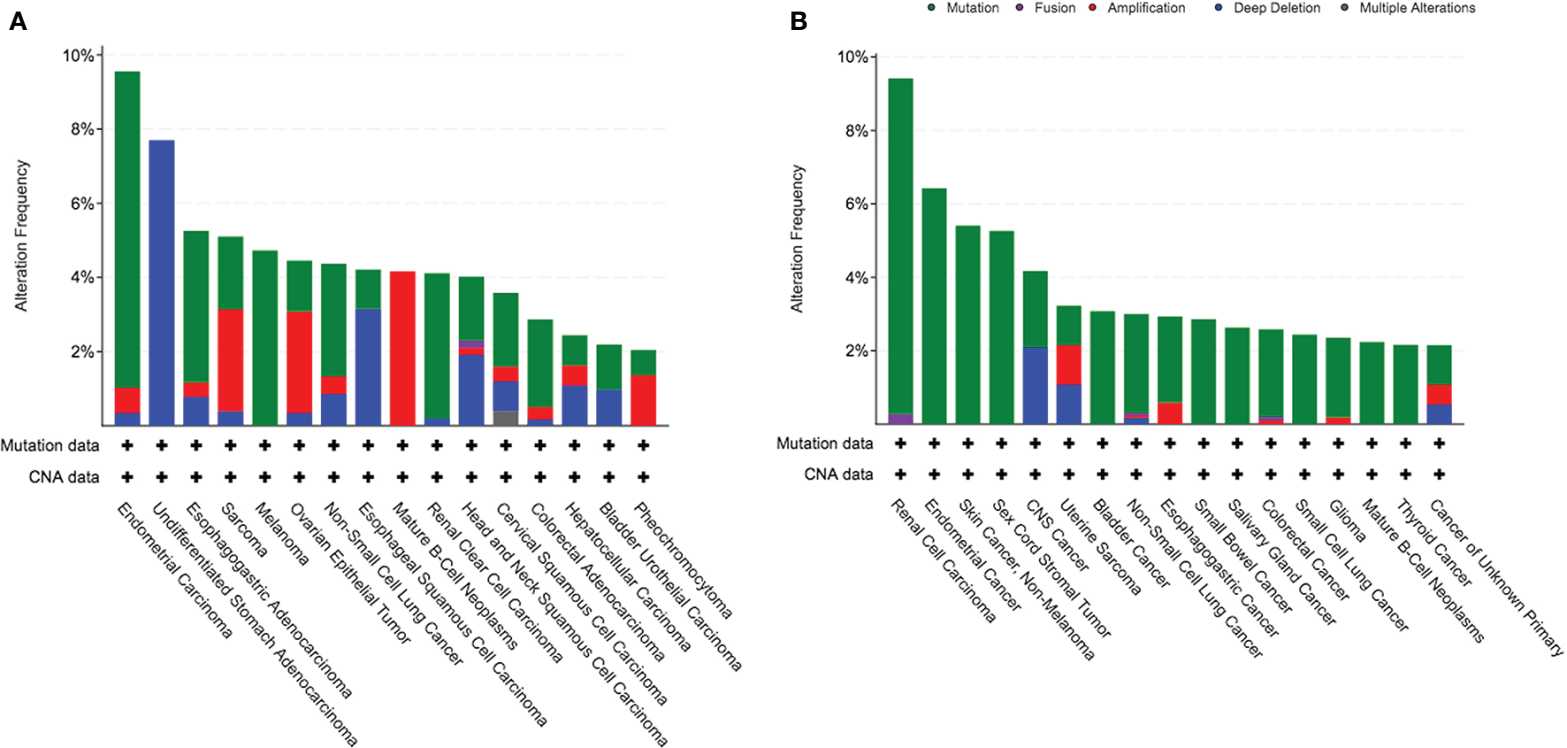
Prevalence of *KDM5C* alterations in different cancers. **(A)** TCGA cohort; **(B)** MSK-IMPACT cohort.

We identified a cohort of 45614 cancer patients with 48045 sequenced tumor samples. This cohort was consisted of 271 cancer studies and 47 cancer types. The prevalence of *KDM5C* alterations was 2.1%, with patients with esophagogastric cancer having the highest levels of *KDM5C* alterations (11.5%, 118/1023). We then investigated the prevalence and spectrum of *KDM5C* alterations in two representative cohorts (TCGA cohort, N = 10967; MSK-IMPACT cohort, N = 10945). In TCGA cohort, endometrial carcinoma had the highest levels of *KDM5C* alterations (9.6%, 56/586; **Figure 2A**). In MSK-IMPACT cohort, renal cell carcinoma had the highest levels of *KDM5C* alterations (9.4%, 34/361; **Figure 2B**). Most detected *KDM5C* alterations were copy number alterations (either amplifications or deep deletion) in TCGA cohort (**Figure 2A**), while most were *KDM5C* somatic mutations in MSK-IMPACT cohort (**Figure 2B**).

### Association Between *KDM5C* Alterations and Clinical Outcomes

**Figure 3:**
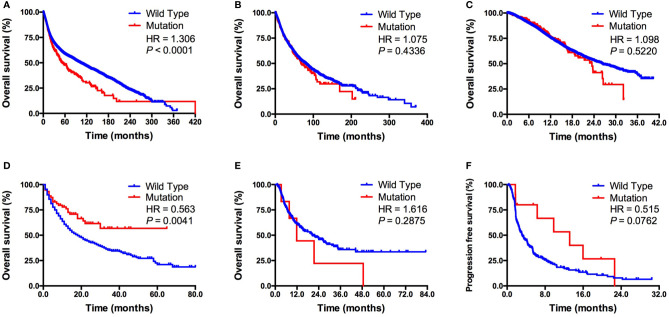
Association between *KDM5C* alterations and clinical outcome. **(A)** Prognostic value of *KDM5C* alterations in all cancers; **(B)** Prognostic value of *KDM5C* alterations in TCGA cohort; **(C)** Prognostic value of *KDM5C* alterations in MSK-IMPACT cohort; **(D)** Predictive value of *KDM5C* alterations in ICI treated cohort; **(E)** Predictive value of *KDM5C* alterations in patients with microsatellite-stable solid tumors; **(F)** Predictive value of *KDM5C* alterations in patients with non-small-cell lung cancer patients received ICI treatment.

Next, we evaluated the association between *KDM5C* alterations and clinical outcomes. We firstly found that patients with *KDM5C* alterations showed a significantly shorter OS (53 vs. 102 months; HR = 1.31, 95% CI 1.17-1.58, *P* < 0.0001; **Figure 3A**) than those without in 45614 cancer patients by merging 271 non-redundant studies from the cBioPortal online database. Subgroup analyses showed that *KDM5C* alterations were correlated with numerically shorter OS in TCGA (68 vs. 80 months; *P* = 0.4336; **Figure 3B**) and MSK-IMPACT cohort (23 vs. 26 months, *P* = 0.5220; **Figure 3C**).

In the ICI treatment cohort (19), we firstly identified 1661 patients with different cancers receiving ICI therapy and 73 of them with *KDM5C* alterations. Clinicopathological features, including age, sex, sample type, drug type and tumor purity, were well balanced between altered and wild type group (**Supplemental Table S1**). Patients with *KDM5C* alterations had a significantly prolonged OS than those in wild-type group (not reached vs. 18 months; HR = 0.56, 95% CI 0.46-0.86, *P* = 0.0041; **Figure 3D**). Importantly, we compared the overall survival of patients who received ICI with those who did not in KDM5C mutant group. As shown in the following figure A, we found that patients received ICI treatment had markedly longer overall survival than those received chemotherapy in KDM5C mutant group (HR = 0.584, P = 0.0168; **Supplemental Figure S2A**). However, in KDM5C wild type group, patients received ICI treatment had analogous overall survival with those received chemotherapy (HR = 0.949, P = 0.1067; **Supplemental Figure S2B**). Although *KDM5C* alterations were associated with higher level of TMB and mutation count, multivariate analysis revealed that *KDM5C* alterations was associated with substantially longer OS than wild type independent of TMB (HR = 0.60, 95% CI 0.40-0.91, *P* = 0.015; **Supplemental Table S2**). Notably, we did not observe the association between *KDM5C* alterations and better OS in patients with microsatellite-stable (MSS) solid tumors (12 vs. 21 months; HR = 1.62, 95% CI 0.50-5.63, *P* = 0.2875; **Figure 3E**). Interestingly, in non-small-cell lung cancer (NSCLC) treated with ICI, patients with *KDM5C* alterations had markedly longer progression-free survival (PFS) than other alterations and wild type groups (13.2 vs. 3.2 months; HR = 0.52, 95% CI 0.34-1.05, *P* = 0.2875; *P* = 0.0762; **Figure 3F**).

### Association Between *KDM5C* Alteration and TMB Level

**Figure 4:**
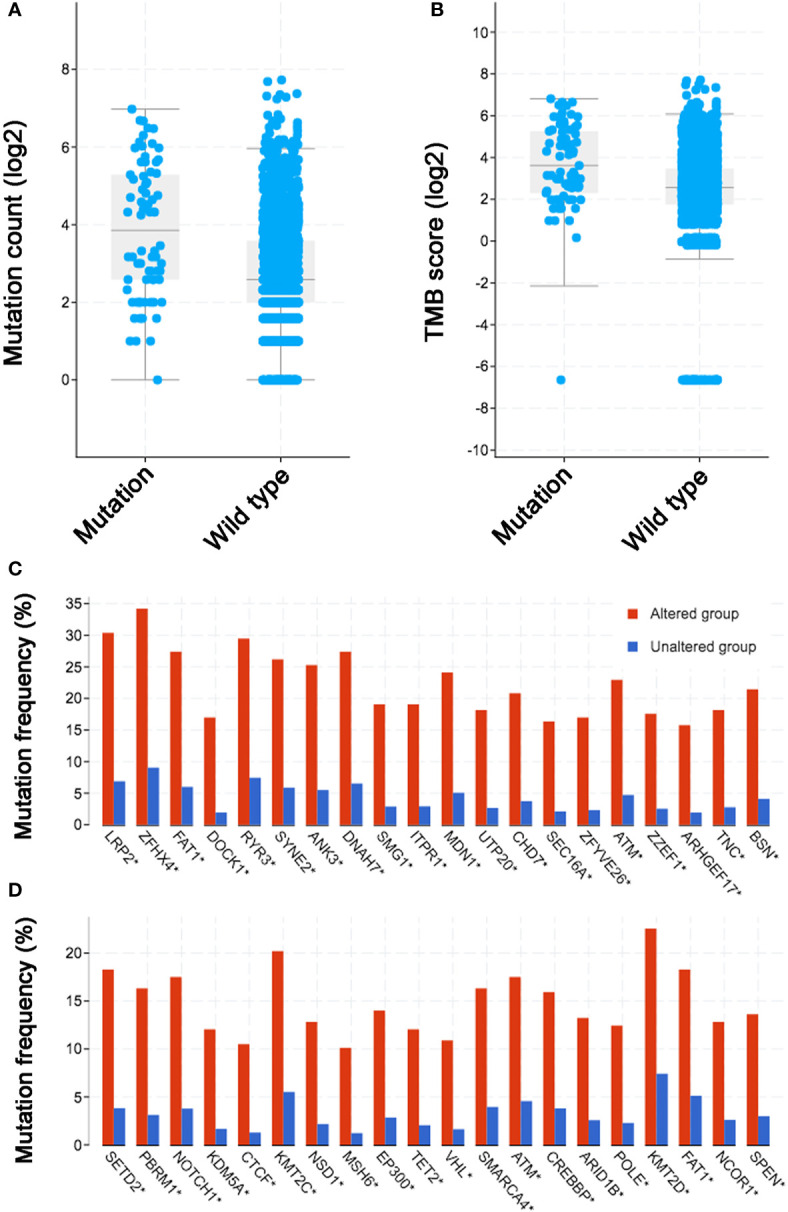
Association between *KDM5C* alterations and mutation count/tumor mutation burden (TMB) across diverse types of cancer. **(A)** The association between mutation count and *KDM5C* alterations in immune checkpoint inhibitor (ICI) treated cohort; **(B)** The association between TMB and *KDM5C* alterations in ICI treated cohort; **(C)** Co-occurring of genetic mutations in cancers with *KDM5C* alterations versus wild type in TCGA cohort; **(D)** Co-occurring of genetic mutations in cancers with *KDM5C* alterations versus wild type in MSK-IMPACT cohort.

Previous publications revealed the close relationship between ICIs treatment outcomes and TMB/mutation counts. Thus, it is valuable to evaluate the relationship between *KDM5C* alterations and TMB level/mutation counts. In MSK-IMPACT cohort (20), we found that mutation count of patients with *KDM5C* alterations was significantly higher than those without these alterations (10 vs. 4, *P* < 0.0001; **Supplemental Figure S1**). This was validated in the ICI-treated cohort that included 1661 patients (mutation count of *KDM5C* alterations vs. wild type: 15 vs. 6, *P* < 0.0001; **Figure 4A**). Notably, cancers with *KDM5C* alterations also had the higher TMB level than those without these alterations (12 vs. 6 mut/Mb, *P* < 0.0001; **Figure 4B**). Co-occurring of genetic mutations in cancers with *KDM5C* alterations were not uncommon in both early-stage and advanced stage cohort (**Figures 4C, D**) and some of them are prevalent driver genes (e.g., *LRP2*, *KMT2C, PBRM1, NOTCH1, FAT1, SETD2, NSD1*, etc.), while their clinical significance remained undetermined.

### Immune Feature Analysis of *KDM5C* Altered Tumors

**Figure 5:**
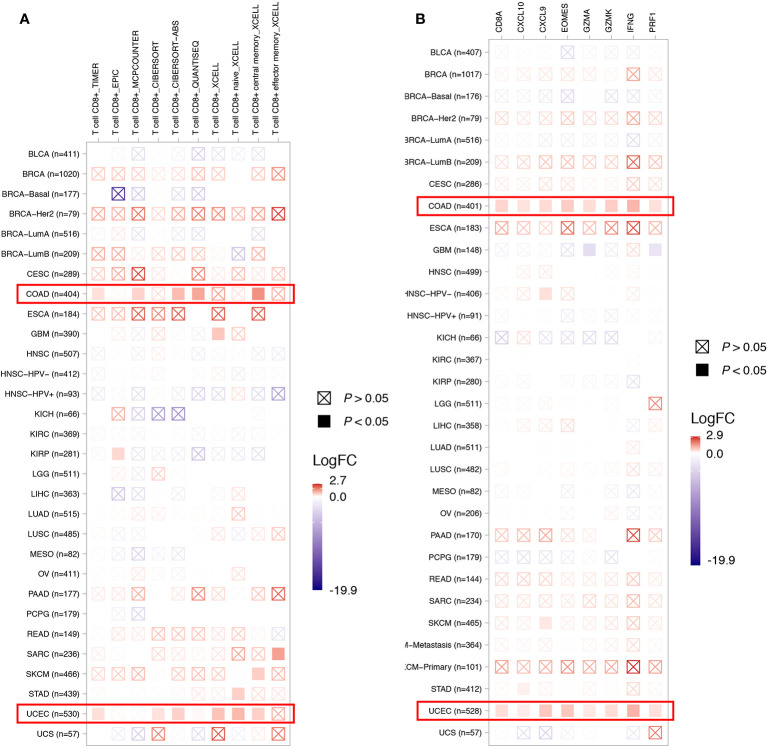
Association between *KDM5C* alterations and CD8+ T cell abundance **(A)** and T effector signature **(B)**. BLCA, Bladder Urothelial Carcinoma; BRCA, Breast invasive carcinoma; CESC, Cervical squamous-cell carcinoma and endocervical adenocarcinoma; COAD, Colon adenocarcinoma; ESCA, esophageal carcinoma; GBM, Glioblastoma multiforme; HNSC, head and neck squamous cell carcinoma; KICH, kidney chromophobe; KIRC, kidney renal clear cell carcinoma; KIRP, kidney renal papillary cell carcinoma; LIHC, Liver hepatocellular carcinoma; LUAD, lung adenocarcinoma; LUSC, lung squamous-cell carcinoma; MESO, mesothelioma; OV, ovarian serous cystadenocarcinoma; PAAD, pancreatic adenocarcinoma; PCPG, pheochromocytoma and paraganglioma; READ, rectum adenocarcinoma; SKCM, Skin cutaneous melanoma; STAD, Stomach adenocarcinoma; UCEC, Uterine corpus endometrial carcinoma; UCS, uterine carcinosarcoma.

**Figure 6:**
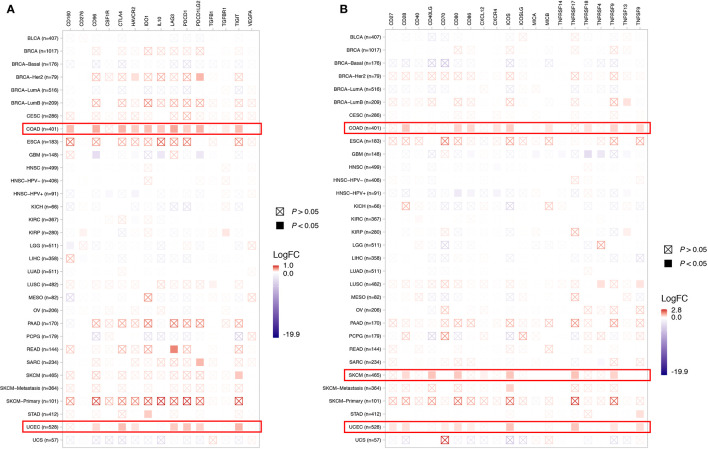
Association between *KDM5C* alterations and several inhibitory **(A)** and stimulatory **(B)** immune checkpoints expression across different cancer types. BLCA, Bladder Urothelial Carcinoma; BRCA, Breast invasive carcinoma; CESC, Cervical squamous-cell carcinoma and endocervical adenocarcinoma; COAD, Colon adenocarcinoma; ESCA, esophageal carcinoma; GBM, Glioblastoma multiforme; HNSC, head and neck squamous cell carcinoma; KICH, kidney chromophobe; KIRC, kidney renal clear cell carcinoma; KIRP, kidney renal papillary cell carcinoma; LIHC, Liver hepatocellular carcinoma; LUAD, lung adenocarcinoma; LUSC, lung squamous-cell carcinoma; MESO, mesothelioma; OV, ovarian serous cystadenocarcinoma; PAAD, pancreatic adenocarcinoma; PCPG, pheochromocytoma and paraganglioma; READ, rectum adenocarcinoma; SKCM, Skin cutaneous melanoma; STAD, Stomach adenocarcinoma; UCEC, Uterine corpus endometrial carcinoma; UCS, uterine carcinosarcoma.

To depict the tumor immune microenvironment of *KDM5C* altered tumors, we compared the immune infiltrates and anti-tumor immunity between *KDM5C* altered and wild type tumors. As we previously mentioned, *KDM5C* altered tumors had significantly higher TMB level than those with wild type, suggesting the potential enhanced tumor immunogenicity of *KDM5C* altered tumors. We then surveyed the relationship between *KDM5C* alterations and common immune infiltrates including CD8^+^ T cells, CD4^+^ T cells, Tregs, dendritic cells, B cells, macrophage, MDSC, NK cells, mast cell, neutrophils, endothelial cells and CAFs across different cancer types (**Figure 5** and **Supplemental Figures S3–S8**). The results showed that tumor-infiltrating CD8^+^ T cells, were generally more abundant in the *KDM5C* altered colon adenocarcinoma and uterine corpus endometrial carcinoma when compared with those in the wild type tumors (**Figure 5A**). Whereas other immune infiltrates had similar abundance in *KDM5C* altered and wild type group (**Supplemental Figures S3–S8**). Moreover, *KDM5C* altered colon adenocarcinoma and uterine corpus endometrial carcinoma had dramatically higher level of antitumor T effector signature (**Figure 5B**). We also evaluated the association between *KDM5C* expression and several inhibitory (e.g., CD160, CD96, CSF1R, CTLA-4, TIM-3, IDO1, IL10, LAG3, PD-1, PD-L1, PD-L2, TFGB1, TGFBR1, TIGIT, VEGFA) and stimulatory (e.g., CD27, CD28, CD40, CD40LG, CD70, CD80, CD86, CXCL12, CXCR4, ICOS, ICOSLG, MICA, MICB, TNFRSF14, TNFRSF17, TNFRSF18, TNFRSF4, TNFRSF9, TNFSF9, TNFSF13) immune checkpoints expression in various cancers. Intriguingly, we also found the significantly higher expression level of these immune checkpoints in *KDM5C* altered colon adenocarcinoma and uterine corpus endometrial carcinoma (**Figures 6A, B**).

## Discussion

To our knowledge, this study firstly reported the frequency of *KDM5C* alterations and its pan-cancer predictive value to ICI treatment in various cancers. *KDM5C* alterations were a negative prognostic marker in whole group but it might be utilized to predict survival benefit from ICI treatment across diverse cancers. Although *KDM5C* altered tumors had significantly higher TMB level, multivariate analysis showed that *KDM5C* alterations was associated with significantly longer OS independent of TMB. Moreover, we did not observe the association between *KDM5C* alterations and prolonged OS in patients with MSS solid tumors, suggesting that it may not be suitable for predicting ICI treatment outcome in MSS solid tumors. Mechanistically, *KDM5C* altered tumors was found to be markedly correlated with enhanced tumor immunogenicity and immunosupportive features of anti-tumor microenvironment.

In this pan-cancer analysis, the frequency of *KDM5C* alterations was 2.1% in a cohort of 45614 cancer patients, with esophagogastric cancer, endometrial carcinoma and renal cell carcinoma having the highest levels of *KDM5C* alterations, which was similar to previous publications (21, 22). Interestingly, we found a positive association between co-occurrence of *KDM5C* alterations and some common epigenetic regulatory genes including *PBRM1, KMT2C, SETD2, NSD1*, etc. In spite of the unclear biological function of these co-mutations, several previous studies have shown that these diver genes are very important tumor suppressor genes in renal cell cancers and could contribute to the aggressive phenotype, therapeutic efficacy and/or prognostic value (23–29). Therefore, it would be valuable to unravel the biological and molecular mechanisms, and impact on clinical outcome of this co-occurrence for specific cancer types in future studies.

Previous studies reported *KDM5C* is required for proper DNA replication at early origins and its alterations could lead to genomic instability in sporadic renal cancer (30, 31). We thus evaluated the association between *KDM5C* alteration and TMB level. As expected, our results showed that *KDM5C* altered tumors had significantly higher TMB level than wild type ones in two independent cohorts, indicating that *KDM5C* alterations could be considered as predictive biomarkers for ICI treatment. Having noticed this relationship, we then investigated both predictive and prognostic significance of *KDM5C* alterations. In whole group, patients with *KDM5C* alterations had a significantly shorter OS than those with wild type, suggesting that *KDM5C* alterations could not confer an intrinsic survival benefit to treatment-naïve patients receiving ICI treatment. In ICI-treated cohort, patients with *KDM5C* alterations had a substantially prolonged OS. Moreover, subgroup analyses showed the association between *KDM5C* alterations and OS was independent of TMB in patients receiving ICI. More interestingly, in NSCLC treated with ICI, we found patients with *KDM5C* alterations had the significantly longer PFS than wild type groups. Collectively, *KDM5C* alterations could be considered as a potential pan-cancer predictive biomarker for ICI treatment, especially for NSCLC.

As a histone demethylase, *KDM5C* could suppress gene transcription by reducing H3K4 trimethylation levels (9–11). *KDM5C* plays a significant role in the tumorigenesis, cancer cell proliferation, invasion, metastasis and drug resistance (12–14). Recently, an elegant study analyzed the multi-omics data of 823 advanced renal cell carcinoma and found that somatic mutations in *KDM5C* correlate with high angiogenesis and AMPK/fatty acid oxidation gene expression, which was enriched in ICIs beneficial group (32). These findings suggested that *KDM5C* altered tumor would have specific immune microenvironment features. In this study, we observed that *KDM5C* altered tumors had markedly higher TMB and were associated with anti-antitumor immune signatures, indicating that *KDM5C* altered tumors would possess the enhanced tumor immunogenicity and relatively immunosupportive microenvironment, supporting its predictive value to ICI treatment.

Pan-cancer universality of immunotherapy targeting PD-1 and PD-L1 interaction challenges us to rethink the investigation and development of predictive biomarkers. To date, MSI-high (MSI-H) is the only pan-cancer biomarker approved by the FDA with a relatively low frequency (~4%) (33, 34). MSI-H is common in digestive cancer including colorectal cancer and gastric cancer, while *KDM5C* alterations were more common in endometrial and renal cell carcinoma, indicating the predictive value of MSI-H and *KDM5C* alterations is not overlapped. Notably, *KDM5C* alterations could not predict the clinical outcome in patients with MSS solid tumors receiving ICI, which need future investigation. Collectively, the pan-cancer predictive significance of *KDM5C* alterations and its complementation to MSI-H in ICI therapy are anticipated.

There are several limitations that should be acknowledged. First, the origin of included cohorts was diverse, which could result in the selection bias and inconsistency of data quality. Combining different groups of patients with distinct histologies without meta-analysis could lead to the methodological pitfalls. Second, the *KDM5C* altered cohort included both gain (e.g., amplifications) and loss (e.g., deletions) of function alterations whether they could cause the same survival or ICI response difference compared to WT remained undetermined. Without adjustment per histology and type of *KDM5C* alterations, these results should be interpreted with caution. Third, due to the unavailable PD-L1 expression results from online database, we could not evaluate the relationship between *KDM5C* alterations and PD-L1 expression. Last but not least, in patients with MSS tumors, only six patients had *KDM5C* alterations. The association between *KDM5C* alterations and prolonged OS in MSS tumors needs further exploration.

In summary, the present study firstly provides the evidence that *KDM5C* alterations were associated with enhanced tumor immunogenicity and inflamed anti-tumor immunity, which result in prolonged OS in cancer patients treated with ICIs. The predictive value of *KDM5C* alterations were independent of tumor mutational burden and microsatellite status, suggesting that *KDM5C* alterations could be considered as a potential pan-cancer predictive biomarker for ICI treatment. In the future, we still need to investigate the exact molecular mechanism and large-scale, prospective studies are also warranted.

## Data Availability Statement

Publicly available datasets were analyzed in this study. This data can be found here: https://www.cbioportal.org.

## Ethics Statement

Ethical approval was waived since we used only publicly available data and materials in this study. The patients/participants provided their written informed consent to participate in this study.

## Author Contributions

X-JC and LZ designed this study. X-JC and AR collected the clinical and sequenced data. X-JC performed the statistical analyses. X-JC, LZ and E-DZ drafted the manuscript. LZ and E-DZ provided critical comments, suggestions and revised the manuscript. All authors contributed to the article and approved the submitted version.

## Conflict of Interest

The authors declare that the research was conducted in the absence of any commercial or financial relationships that could be construed as a potential conflict of interest.
