# Supplementary material for: Predictive Value of KDM5C Alterations for Immune Checkpoint Inhibitors Treatment Outcomes in Patients With Cancer
Source: Front Immunol. 2021 Apr 19;12:664847. doi: 10.3389/fimmu.2021.664847 (PMC8089485; doi:10.3389/fimmu.2021.664847)
Supplement: Supplementary file 1 [file DataSheet_1.docx]

**Supplemental Materials**

**Predictive value of KDM5C alterations for immune checkpoint inhibitors treatment outcomes in patients with cancer**

Supplemental text…………………………………………...…………………..2

Supplemental Figure S1………………………………………………………..4

Supplemental Figure S2………………………………………………………..5

Supplemental Figure S3………………………………………………………..6

Supplemental Figure S4………………………………………………………..7

Supplemental Figure S5………………………………………………………..8

Supplemental Figure S6………………………………………………………..9

Supplemental Figure S7………………………………………………………..10

Supplemental Figure S8………………………………………………………..11

Supplemental Table S1…………………………………………………………12

Supplemental Table S2………………………………………………………....13

References…………………………………………...………………………......14

**Supplemental text**

**TMB normalization and calculation**

Similar to previous study(1), TMB was defined as the total number of nonsynonymous mutations including somatic, coding, base substitution, and indel mutations per megabase (mut/Mb) of genome examined. Mutations in driver oncogenes were also recorded. To evaluate the difference of TMB level between *TERT* altered and *TERT* wild type group, a subset generated from MSK-IMPACT cohort was selected to avoid the selection bias and ensure the TMB could be comparable(2). Then, TMB distributions of *TERT* altered and wild type group were calculated by using an ICI-treated cohort including 1661 patients(3). For samples identified from MSK-IMPACT cohort, the total number of nonsynonymous mutations was normalized to the exonic coverage of the MSK-IMPACT panel (0.98, 1.06, and 1.22 Mb in the 341-, 410-, and 468-gene panels, respectively). For the ICI-treated cohort, TMB was defined as the total number of somatic mutations, which was normalized to the exonic coverage of the respective MSK-IMPACT panel.

**Clinical cohort and treatment outcome**

To investigate the predictive and prognostic significance of *TERT* alterations and its specific subtypes, clinical outcomes and mutational sequencing data from total patients and those receiving ICI treatment were collected. Firstly, we analyzed the predictive and prognostic significance of *TERT* alterations in all included patients with various tumors. Subgroup analyses were further conducted to explore its value in early-stage (TCGA cohort) and advanced stage cancers (MSK-IMPACT cohort), respectively. Then, two independent ICI-treated cohort including multiple cancer types were used to explore the predictive value of *TERT* alterations for ICIs treatment outcomes(3, 4). Subgroup analyses were also performed to explore the predictive value of *TERT* alteration subtypes. We also surveyed the relationship between *TERT* alterations and treatment response to ICIs in NSCLC(5), one of the most common solid tumors and leading cause of cancer-related death worldwide. Finally, the predictive value of *TERT* alterations and its subtypes were further validated by using our real-world cohort from three medical centers. Response including complete response, partial response, stable disease and disease progression (PD) was assessed using Response Evaluation Criteria in Solid Tumors version 1.1. Progression-free survival was assessed from the date the patient began ICI treatment to the date of PD or death of any cause. Patients who had not progressed were censored at the date of their last follow-up. Overall survival (OS) was calculated from the beginning of immunotherapy to the date of death of any cause. Patients who was still alive or lost contact were censored at the date of last scan. Notably, in the non-ICI-treated cohort from TCGA cohort, OS was defined as the time between first diagnosis and death or last follow-up. In MSK-IMPACT cohort, OS was calculated from the date of the procedure date when the tumor specimen was collected to death or last follow-up(2). This study was conducted in accordance with the provisions of the Declaration of Helsinki and was approved by the ethics committee of each medical center.

**
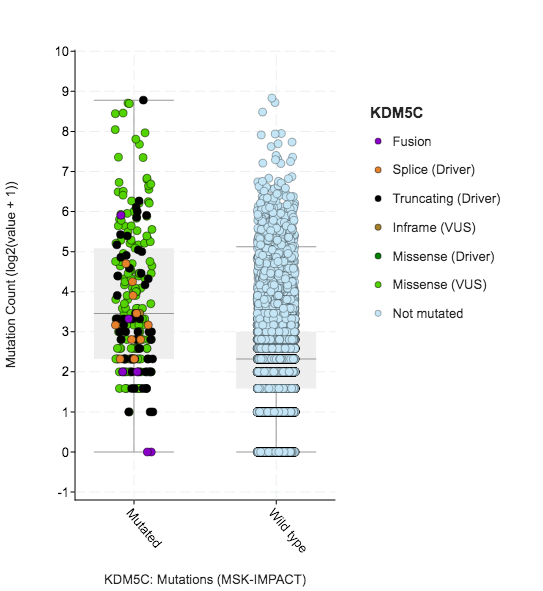
**

**Supplemental Figure S1. The association between mutation count and *KDM5C* alterations in MSK-IMPACT cohort.**


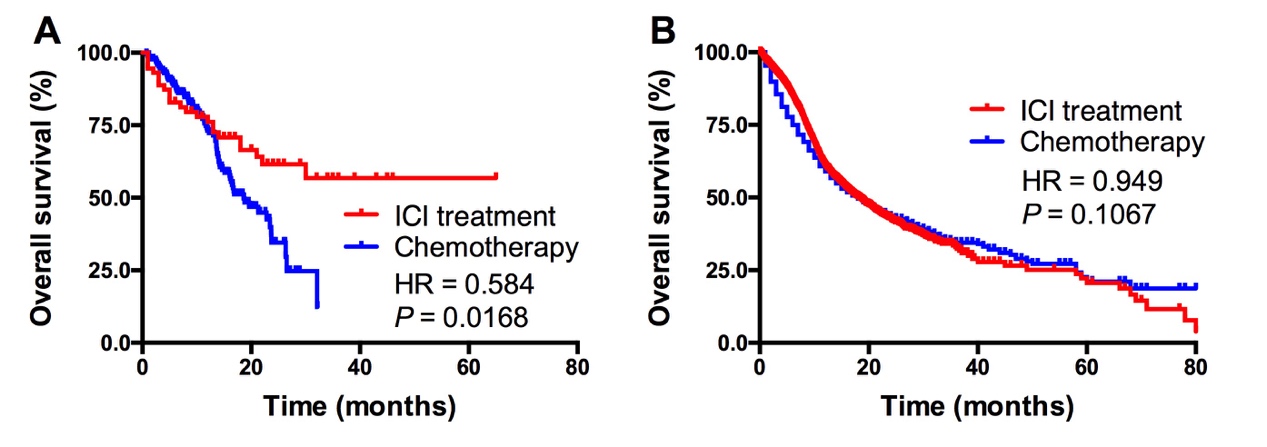


**Supplemental Figure S2. Comparison of overall survival between patients who received ICI versus those who received chemotherapy in KDM5C mutant group (A) and wild type group (B), respectively.**

**Supplemental Figure S3. Comparison of CD4+ T cells abundance between *KDM5C* alteration and wild type group.**

**Supplemental Figure S4. Comparison of B cells abundance between *KDM5C* alteration and wild type group.**

**Supplemental Figure S5. Comparison of dendritic cells abundance between *KDM5C* alteration and wild type group.**

**Supplemental Figure S6. Comparison of NK, mast cells and neutrophils abundance between *KDM5C* alteration and wild type group.**

**Supplemental Figure S7. Comparison of Macrophage, regulatory T cells and MDSC abundance between *KDM5C* alteration and wild type group.**

**Supplemental Figure S8. Comparison of endothelial cells and cancer-associated fibroblasts abundance between *KDM5C* alteration and wild type group.**

| **Supplemental Table S1. Baseline characteristics of the study population.** | | | | | | | |
| --- | --- | --- | --- | --- | --- | --- | --- |
| **Variables** | **All cases** |  | ***KDM5C* alterations** | | **KDM5C wild type** | | ***P* value** |
| Total | 1661 |  | 73 | 4.39% | 1588 | 95.61% |  |
| Age at diagnosis |  |  |  |  |  |  |  |
| < 65 years | 922 |  | 44 | 4.77% | 878 | 95.23% | 0.402 |
| ≥ 65 years | 738 |  | 29 | 3.93% | 709 | 96.07% |  |
| NA | 1 |  | 0 | 0.00% | 1 | 100.00% |  |
| Gender |  |  |  |  |  |  |  |
| Male | 627 |  | 23 | 3.67% | 604 | 96.33% | 0.261 |
| Female | 1034 |  | 50 | 4.84% | 984 | 95.16% |  |
| Sample type |  |  |  |  |  |  |  |
| Primary | 930 |  | 39 | 4.19% | 891 | 95.81% | 0.652 |
| Metastasis | 731 |  | 34 | 4.65% | 697 | 95.35% |  |
| Drug type |  |  |  |  |  |  |  |
| PD-1/PDL-1 | 1307 |  | 63 | 4.82% | 1244 | 95.18% | 0.162 |
| CTLA4 | 99 |  | 3 | 3.03% | 96 | 96.97% |  |
| Combo | 255 |  | 7 | 2.75% | 248 | 97.25% |  |
| Tumor purity |  |  |  |  |  |  |  |
| < 50 | 826 |  | 31 | 3.75% | 795 | 96.25% | 0.131 |
| ≥ 50 | 770 |  | 41 | 5.32% | 729 | 94.68% |  |
| NA | 65 |  | 1 | 1.54% | 64 | 98.46% |  |
| Mutation count |  |  |  |  |  |  |  |
| Median (range) | 6 (1-212) |  | 15 (1-126) | | 6 (1-212) | | < 0.001 |
| TMB score |  |  |  |  |  |  |  |
| < 10 | 1173 |  | 36 | 3.07% | 1137 | 96.93% | < 0.001 |
| ≥ 10 | 488 |  | 37 | 7.58% | 451 | 92.42% |  |
| TMB, tumor mutation burden; NA, not applicable. | | | | | | | |

| **Supplemental Table S2. Multivariate analyses of clinical parameters on overall survival.** | | | |
| --- | --- | --- | --- |
| **Factor** | **HR (log rank)** | **95% CI** | ***P* value** |
| Sex (Female/male) | 1.110 | 0.965-1.277 | 0.142 |
| Age (<65/>65) | 0.998 | 0.869-1.147 | 0.983 |
| Therapy (combo/mono) | 0.552 | 0.445-0.684 | <0.001 |
| Tumor purity(>50/<50) | 0.902 | 0.786-1.035 | 0.142 |
| TMB score (>10/<10) | 0.554 | 0.458-0.670 | <0.001 |
| Mutation count (>6/<6) | 0.927 | 0.792-1.086 | 0.345 |
| *KDM5C* (alteration/wild type) | 0.602 | 0.400-0.905 | 0.015 |
| mono, monotherapy; combo, combination therapy; TMB, tumor mutation burden. | | | |

**References**

1. Yarchoan M, Hopkins A and Jaffee EM. Tumor Mutational Burden and Response Rate to PD-1 Inhibition. *N Engl J Med* 2017;377:2500-2501.

2. Zehir A, Benayed R, Shah RH, et al. Mutational landscape of metastatic cancer revealed from prospective clinical sequencing of 10,000 patients. *Nat Med* 2017;23:703-713.

3. Samstein RM, Lee CH, Shoushtari AN, et al. Tumor mutational load predicts survival after immunotherapy across multiple cancer types. *Nat Genet* 2019;51:202-206.

4. Miao D, Margolis CA, Vokes NI, et al. Genomic correlates of response to immune checkpoint blockade in microsatellite-stable solid tumors. *Nat Genet* 2018;50:1271-1281.

5. Rizvi H, Sanchez-Vega F, La K, et al. Molecular Determinants of Response to Anti-Programmed Cell Death (PD)-1 and Anti-Programmed Death-Ligand 1 (PD-L1) Blockade in Patients With Non-Small-Cell Lung Cancer Profiled With Targeted Next-Generation Sequencing. *J Clin Oncol* 2018;36:633-641.
